# Editorial: Computational morphofunctional assessment of coronary artery disease

**DOI:** 10.3389/fcvm.2024.1472518

**Published:** 2024-09-20

**Authors:** Jelmer Westra, Shengxian Tu, Linghong Shen

**Affiliations:** ^1^ Department of Cardiology, Linköping University Hospital, Linköping, Sweden; ^2^ Biomedical Instrument Institute, School of Biomedical Engineering, Shanghai Jiao Tong University, Shanghai, China; ^3^ Department of Cardiology, Shanghai Chest Hospital, Shanghai Jiaotong University School of Medicine, Shanghai, China

**Keywords:** coronary artery disease, computational morphofunctional assessment, intravascular imaging, computed tomography angiography, quantitative flow ratio

**Editorial on the Research Topic**
Computational morphofunctional assessment of coronary artery disease

## Main test

Identification of ischemia-producing lesions through functional assessment is fundamental in the treatment of patients with chronic coronary syndrome when percutaneous coronary intervention is considered. This process often includes evaluation of hemodynamic significance of obstructive epicardial coronary stenosis as well as microvascular disease without obstructive epicardial stenosis (1). In addition to functional evaluation, morphological assessment of coronary artery disease has gained traction for planning and evaluation of invasive procedures (2).

The growing availability of computational solutions allows for an comprehensive assessment of coronary artery disease (3). This special issue of Frontiers Cardiovascular Medicine on “Computational Morphofunctional assessment of Coronary Artery Disease” provides new insight into advancements of identifying clinical scenarios where computational morphofunctional modelling can assist prognostication and clinical decision-making. In total, four papers were included and summarized below.

Ágoston et al. present a proof-of-concept study won the derivation of microvascular resistance reserve (MRR) using solely intracoronary pressure data (MRRpb) and hemodynamic modelling using quantitative coronary angiography (MRRp-3D). The predicted MRR values correlated (*r *= 54) and agreed (mean difference 0.04 ± 0.88) with MRR derived using the Doppler technique.

Hu et al. provide further evidence underlying the clinical value of estimating physiology from intravascular imaging data using optical coherence tomography (optical flow ratio, OFR). In a study including 354 patients that underwent OCT-guided PCI, post-PCI OFR [HR 0.60 (95%CI: 0.41–0.89) per 0.1 increase in OFR] was independently associated with target vessel failure, alongside large stent edge detection and thin-cap fibroatheroma after a median follow-up of 484 days.

Radunović et al. timely assessed the ability of coronary computed tomography angiography (CTA) to evaluate true bifurcation lesions. The study found that coronary CTA-based assessment of vessel diameters and plaque density correlated acceptably to intravascular ultrasound derived estimates. This study highlights the potential for procedural planning based on coronary CTA as an integrated solution in the catheterization laboratory, pending further validation.

Xu et al. examined the association between post-PCI side-branch Murray-based quantitative flow ratio (uQFR) with side-branch coronary blood flow at 6 and 24 months, as assessed with TIMI flow grade. They found that the incidence of side-branch TIMI flow grade <1 and <2 at follow-up decreased according to post-procedure uQFR tertiles. It confirmed the association between post-procedural SB μQFR and long-term SB coronary blood ﬂow in non-LM coronary bifurcation lesions receiving one-stent strategy.

The presented manuscripts underscore the progress in imaging for interventional cardiology, particularly with the use of computational morphofunctional assessment across different imaging modalities. However, further research is needed to evaluate the feasibility and clinical applicability of these methods in routine clinical practice.
